# Safety and efficacy of unilateral and bilateral pallidotomy for primary dystonia

**DOI:** 10.1002/acn3.51333

**Published:** 2021-03-15

**Authors:** Shiro Horisawa, Atsushi Fukui, Nobuhiko Takeda, Takakazu Kawamata, Takaomi Taira

**Affiliations:** ^1^ Department of Neurosurgery Neurological Institute Tokyo Women’s Medical University Tokyo Japan

## Abstract

**Objective:**

Ablation of the globus pallidus internus (pallidotomy) is an effective surgical intervention for dystonia. However, the current literature on the efficacy and safety of pallidotomy for dystonia is derived only from single‐case reports and small cohort studies.

**Methods:**

We retrospectively analyzed patients with primary dystonia who underwent pallidotomy at our institution between 2014 and 2019. Neurological conditions were evaluated using the Burke‐Fahn‐Marsden Dystonia Rating Scale (BFMDRS, range: 0–120). We evaluated the total BFMDRS score and each subitem score (nine body regions) in the patients who underwent unilateral and bilateral pallidotomy before surgery and at last available follow‐up. Moreover, postoperative complications were analyzed.

**Results:**

We found that 69 and 20 patients underwent unilateral and bilateral pallidotomy respectively. The mean age at dystonia onset was 40.4 ± 15.2 years. The mean clinical follow‐up period was 17.2 ± 11.6 months. Unilateral pallidotomy significantly improved the total BFMDRS score from 11.2 ± 14.7 preoperatively to 5.4 ± 7.6 at last available follow‐up (51.8% improvement, *p* < 0.001). Furthermore, there was a significant and independent improvement in all midline BFMDRS subitems, including eyes, mouth, speech/swallow, and neck, after unilateral pallidotomy. Bilateral pallidotomy significantly improved the total BFMDRS score from 14.6 ± 10.2 preoperatively to 3.8 ± 8.2 at last available follow‐up (74.0% improvement, *p* < 0.001). However, bilateral pallidotomy induced medically refractory parkinsonism (postural instability and gait disturbance) in five patients, dysarthria in three patients, and dysphagia in one patient.

**Interpretation:**

Unilateral radiofrequency pallidotomy remains a viable treatment option for patients with some forms of dystonia. Bilateral pallidotomy cannot be recommended due to unacceptably high complication rates.

## Introduction

Dystonia is a common movement disorder that is characterized by sustained and repetitive movements or abnormal postures.^1^ It develops in every body part and its distribution ranges from focal to generalized dystonia. Conservative dystonia treatments include botulinum toxin injections and oral medications.^2^ However, widely spread dystonia involving cranio‐cervico‐axial regions is often refractory to these conservative treatments.

Deep brain stimulation (DBS) of the globus pallidus internus (GPi) is the most applied surgical treatment for dystonia and has been validated by numerous clinical studies.^3^, ^4^, ^5^ However, several studies have reported a high prevalence (11%–25.3%) of hardware‐related complications associated with DBS surgery.^6^, ^7^, ^8^, ^9^

GPi ablation (pallidotomy) is an alternative surgical option for dystonia that is rarely used in most developed countries where DBS is widely available. The small cohort studies report the efficacies of pallidotomy and GPi‐DBS for dystonia are comparable.^10^, ^11^, ^12^, ^13^, ^14^ Pallidotomy plays an especially important role in dystonia treatment among individuals opposed to device implantation or those unable to access DBS for economic or geographical reasons.^15^ However, the current literature on the efficacy of pallidotomy for dystonia is solely derived from single case reports and small cohort studies. Therefore, the efficacy and safety of pallidotomy for dystonia remains unclear.^16^

In this study, we aimed to perform a detailed assessment of the efficacy and safety of pallidotomy, including bilateral procedures. To the best of our knowledge, this is the largest study to assess the efficacy and safety of pallidotomy for dystonia.

## Patients and Methods

### Methods

This retrospective study was approved by the ethics committee of our institution, which waived the requirement for informed patient consent given the observational nature of this study.

### Patient population

Between 2014 and 2019, 95 consecutive patients with primary dystonia underwent unilateral or bilateral pallidotomy at our institution. The inclusion criteria were a minimum of 1‐year post‐symptom onset and a previous diagnosis of primary dystonia by a neurologist or neurosurgeon specialized in movement disorders. The exclusion criteria were secondary dystonia (drug‐/trauma‐/stroke‐induced dystonia, etc.) and having undergone previous neurosurgical treatments, including other intracranial surgeries such as DBS and selective peripheral denervation. We excluded six patients (5: drug‐induced dystonia, 1: stroke‐induced dystonia) and included 89 patients in the final analysis.

### Surgical procedures

Under local anesthesia, a Leksell stereotactic frame (Elekta, Stockholm, Sweden) was fixed onto the patient’s skull. T1‐weighted axial and T2‐weighted coronal magnetic resonance imaging (MRI) and computed tomography scans were used to determine the stereotactic GPi target. The tentative target was a location 2–4 mm below, 18.5–22 mm lateral, and 2 mm anterior to the midpoint of the anterior and posterior commissures. A radiofrequency probe (1.0 mm diameter with a 4.0 mm uninsulated tip) and Leksell neurogenerator were used for stimulation and coagulation. Surgery was performed under local anesthesia without microelectrode recording. After ensuring the absence of capsular response and phosphene through macrostimulation via the electrode (130 Hz, 100 µsec pulse width, and 5–10 mA), coagulation was performed at 70°C for 30–40 sec. Immediate post‐surgery MRI was used to examine for coagulated lesions and hemorrhagic complications. Detailed information regarding the pallidotomy procedure has been described in previous articles.^17^

### Treatment strategy

All patients except for two patients who underwent simultaneous bilateral pallidotomy, underwent initial unilateral pallidotomy. The surgery site for unilateral pallidotomy was decided based on the dystonia distribution. Generally, the contralateral hemisphere to the most affected side by dystonia was chosen as the surgical site. For torticollis, the contralateral hemisphere to the direction of head deviation was chosen as the surgical side, for example, right‐side rotation–left pallidotomy or left‐side rotation–right pallidotomy. For laterocollis, the contralateral side to the direction of neck tilting was chosen as the surgical side, for example, right laterocollis–left pallidotomy. Based on the patients’ discretion, they underwent contralateral side surgery or continued follow‐up at 6 months after unilateral pallidotomy. Patients seeking further dystonia improvement were scheduled to undergo contralateral side pallidotomy. There was an interval of ≥6 months between the first and second surgery.

### Evaluation procedures

We used the Burke‐Fahn‐Marsden Dystonia Rating Scale (BFMDRS) to evaluate the patients’ dystonic conditions preoperatively and at last available follow‐up. The BFMDRS, which has a score that ranges between 0 (minimum/best) and 120 (maximum/worst), is used to evaluate dystonia severity in 9 body regions, including the eyes (0–8), mouth (0–8), speech/swallow (0–16), neck (0–8), trunk (0–16), right arm (0–16), left arm (0–16), right leg (0–16), and left leg (0–16). We evaluated the total BFMDRS score and each subitem score in patients who underwent unilateral and bilateral pallidotomy. Regarding unilateral pallidotomy evaluation, the subitems for the arms and legs were divided into the contralateral or ipsilateral surgery side. Moreover, we analyzed postoperative complications.

### Statistical analysis

We performed a Shapiro–Wilk normality test to confirm data normality. Since the data were non‐normally distributed, the Wilcoxon signed‐rank test was used to compare the preoperative total and subitem BFMDRS scores with those at last available follow‐up. All statistical analyses were performed using the SPSS 25.0 (SPSS Inc., Chicago, IL, USA). All statistical tests were two‐tailed and statistical significance was set at *p*‐value <0.05.

## Results

Table 1 shows detailed patients’ and demographic characteristics. There were 69 (29 right side: 40; left side) and 20 patients who underwent unilateral and bilateral pallidotomy respectively. The mean age at onset of dystonia was 40.4 ± 15.2 years. The mean clinical follow‐up period was 17.2 ± 11.6 months. The dystonia distribution was as follows: focal dystonia (54 patients), segmental dystonia (24 patients), and generalized dystonia (11 patients). The mean pre‐operative BFMDRS score was 13.8 ± 1.5.

**Table 1:** Patient characteristics.

Number of patients	89
Male	59
Female	30
Age at onset (year)	40.4 ± 15.2
Age at surgery (year)	48.1 ± 14.1
Mean follow‐up period (month)	17.2 ± 11.6
BFMDRS	13.8 ± 1.5
Distribution of dystonia	
Generalized dystonia	11
Segmental dystonia	
Meige syndrome	17
Other segmental dystonia	7
Focal dystonia	
Cervical dystonia	33
Focal arm/hand dystonia	8
Mouth dystonia	7
Tongue dystonia	3
Blepharospasm	2
Spasmodic dysphonia	1
Unilateral pallidotomy	69
Rt	29
Lt	40
Bilateral pallidotomy	20
Simultaneous	2
Staged	18

Data are presented as mean ± standard deviation.

BFMDRS, Burke‐Fahn‐Marsden Dyatonia Rating Scale.

### Unilateral pallidotomy

**Figure 1:**
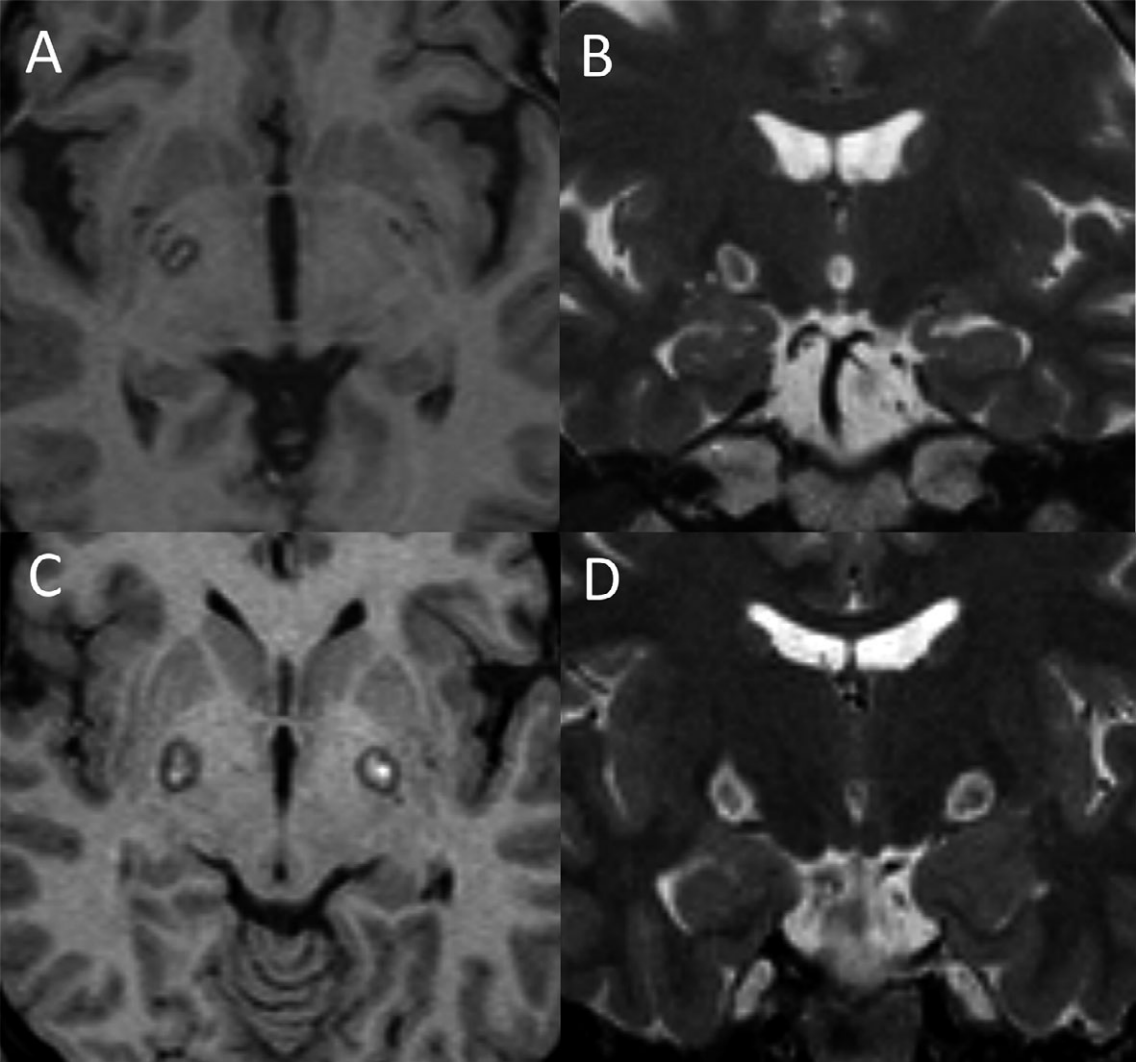
MRI images of the patients after undergoing pallidotomy. (A–B) show postoperative T1‐ and T2‐weighted MRI after undergoing unilateral pallidotomy. (C–D) show postoperative T1‐ and T2‐weighted MRI after undergoing simultaneous bilateral pallidotomy.

There were 29 and 40 patients who underwent right‐side and left‐side pallidotomy, respectively (Fig. 1A and B). The mean follow‐up period was 15.3 ± 10.9 months. The total BFMDRS score significantly improved from 11.2 ± 14.7 preoperatively to 5.4 ± 7.6 at last available follow‐up (51.8% improvement, *p* < 0.001). Arm and leg dystonia in the contralateral side was significantly improved compared with those in the ipsilateral side. There was a significant improvement in all midline BFMDRS subitems, including eyes, mouth, speech/swallow, and neck, after unilateral pallidotomy at the last available follow‐up. Table 2 presents the detailed clinical outcomes.

**Table 2:** Clinical outcomes of unilateral pallidotomy.

		Number of affected patients	Before surgery	Last follow‐up	% improvement	*p* *
BFMDRS	Total	69	11.2 ± 14.7	5.4 ± 7.6	51.80%	<0.001
	Contralateral side					
	Arm	18	7 ± 4.3	2.1 ± 3.4	70.00%	<0.001
	Leg	2	14 ± 2.8	1.5 ± 0.7	89.30%	0.180
	Ipsilateral side					
	Arm	6	9.7 ± 6.0	7.9 ± 5.7	18.60%	0.317
	Leg	2	12 ± 5.7	7 ± 2.6	41.70%	0.317
	Midline					
	Eyes	15	4.5 ± 2.2	2.5 ± 2.5	44.40%	0.0205
	Mouth	18	3.6 ± 2.3	1.7 ± 2.2	52.80%	0.005
	Speech/swallow	17	5.8 ± 3.3	2.3 ± 2.8	60.30%	0.002
	Neck	40	5.4 ± 2.6	2.7 ± 2.5	50.00%	<0.001
	Trunk	10	9.5 ± 4.5	5.4 ± 5.8	43.20%	0.016
Follow‐up period (month)	15.3 ± 10.9					

Data are presented as mean ± standard deviation.

BFMDRS, Burke‐Fahn‐Marsden Dyatonia Rating Scale.

* Wilcoxon signed‐rank test.

### Bilateral pallidotomy

There were 2 and 18 patients who underwent simultaneous and staged bilateral pallidotomy, respectively (Fig. 1C and D). The mean interval between staged surgeries was 8.2 ± 3.8 months. The total BFMDRS score significantly improved from 14.6 ± 10.2 preoperatively to 3.8 ± 8.2 at last available follow‐up (74.0% improvement, *p* < 0.001). Regarding midline dystonia distribution, all subitems other than “trunk,” were significantly improved after bilateral pallidotomy. Table 3 presents the detailed clinical outcomes.

**Table 3:** Clinical outcomes of bilateral pallidotomy.

		Number of affected patients	Before surgery	Last follow‐up	% Improvement	*p* *
BFMDRS	Total	20	14.6 ± 10.2	3.8 ± 8.2	74.00%	<0.0001
	Right side					
	Arm	4	11.3 ± 3.6	0.7 ± 1.2	93.80%	0.068
	Leg	0	–	–		
	Left side					
	Arm	4	8 ± 1.4	2.3 ± 4.5	71.30%	0.109
	Leg	1	12	0	100%	0.317
	Midline					
	Eyes	8	6.3 ± 1.3	3.3 ± 3.4	47.60%	0.026
	Mouth	6	4.3 ± 2.0	2.4 ± 3.3	44.20%	0.039
	Speech/swallow	7	4.0 ± 1.6	1.2 ± 1.6	70.00%	0.026
	Neck	16	5.2 ± 2.0	0.9 ± 1.7	73.10%	<0.001
	Trunk	4	5.0 ± 2.6	1.5 ± 3	70.00%	0.109
Follow‐up period (month)	23.6 ± 12.2					
Interval between staged surgeries (month)	8.2 ± 3.8					

Data are presented as mean ± standard deviation.

BFMDRS, Burke‐Fahn‐Marsden Dyatonia Rating Scale.

* Wilcoxon signed‐rank test.

### Adverse events

**Figure 2:**
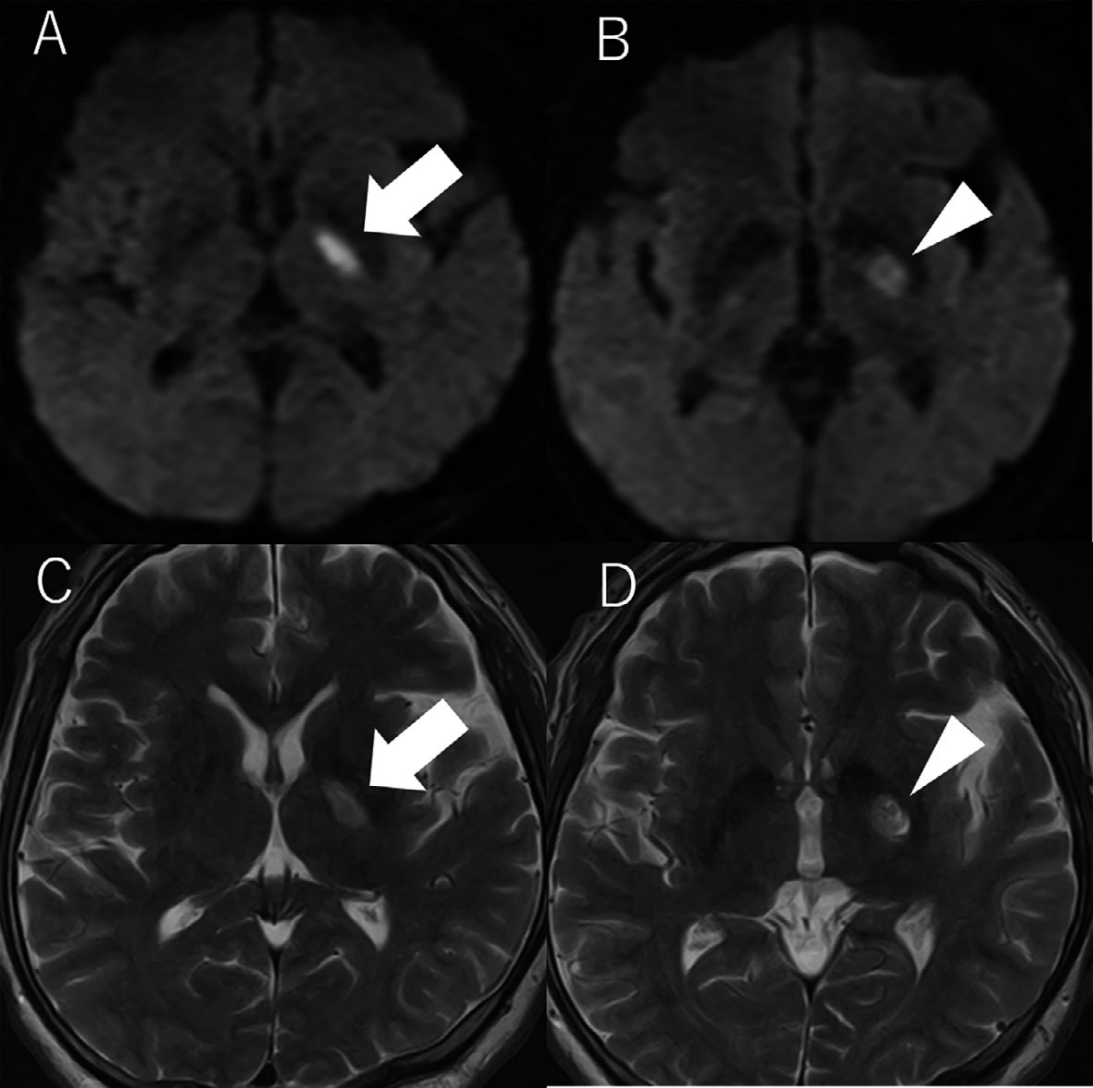
Diffusion‐weighted (A and B) and T2‐weighted (C and D) MRI images of cerebral infarction 1 month after pallidotomy. A and C show acute cerebral infarction on the left posterior limb of internal capsule (arrow). B and D show left pallidotomy lesion (arrowhead).

Table 4 presents the detailed adverse events. Intracerebral hemorrhage occurred in 6 of 109 procedures (5.5%); moreover, all hemorrhage cases were asymptomatic and confirmed only in the site of pallidotomy lesion. Of 109 procedures, five (4.6%) presented with cerebral infarction on the posterior limb of internal capsule, which resulted in temporary hemifacial palsy or hemiparesis (Fig. 2). However, none of the cerebral infarction cases resulted in prolonged neurological deficits. All infarction cases developed between a few weeks and one month after surgery.

**Table 4:** Adverse events.

	Unilateral pallidotomy	Bilateral pallidotomy
Cerebral infarction	3	1
Hemorrhage	5	1
Asymptomatic	0	0
Symptomatic		
Parkinsonism^1^	3	5
Hemiparesis^1^	2	0
Visual disturbance^1^	1	0
Dysarthria^1^	0	4
Dysphagia^1^	0	1

^1^ Adverse events not associated with cerebral infarction and hemorrhage.

Parkinsonism was observed in five (25%) and 2 (2.9%) patients who underwent bilateral and unilateral pallidotomy. Those five patients who underwent bilateral pallidotomy presented with postural instability and gait disturbance; among them, two cases were severe with assistance from another person being required. During the follow‐up period, postural instability and gait disturbance did not improve in all patients. Micrographia was observed in two and three patients who underwent unilateral and bilateral pallidotomy respectively. Oral levodopa intake was attempted but was not effective for all patients. There was a postoperative spontaneous improvement of micrographia for 3–6 months. However, postural instability and gait disturbance persisted in all patients at the last available follow‐up evaluation. Persistent dysarthria and dysphagia were observed in four and one patient respectively. Among four patients presenting permanent dysarthria, one patient had concomitant parkinsonism (postural instability and gait disorder).

## Discussion

This study has three major findings. First, unilateral pallidotomy achieved 51.8% improvement of the total BFMDRS score, which was consistent with the findings of open‐label studies on bilateral GPi‐DBS for dystonia (45%–51% BFMDRS score improvement).^4^, ^5^ Second, unilateral pallidotomy significantly improved all midline BFMDRS subitems (eyes, mouth, speech/swallow, neck, and trunk). Third, bilateral pallidotomy was associated with parkinsonism, including postural instability and gait disturbance, which was medically refractory and could not improve spontaneously.

Pallidotomy for dystonia was commonly used until the 1990s.^18^ In the mid‐1980s, studies reported significant effects of pallidotomy on dyskinesia and dystonia in patients with Parkinson’s disease (PD),^19^ which promoted the use of pallidotomy for patients with primary dystonia.^14^, ^20^, ^21^ However, the advent of GPi‐DBS in the 1990s led to a gradual abandonment of pallidotomy as a dystonia treatment. Within the last decade, only seven patients with primary and secondary dystonia have been reported to undergo pallidotomy for dystonia treatment with our patients excluded.^22^, ^23^, ^24^, ^25^, ^26^, ^27^ This could be attributed to the lack of evidence for pallidotomy.^18^ There has been no randomized controlled study for confirming the safety and efficacy of pallidotomy for dystonia. The current literature on the efficacy of pallidotomy is derived from small case studies and reports using objective evaluating scales such as the BFMDRS or Toronto Western Spasmodic Torticollis Rating Scale (TWSTRS).

Previous studies on bilateral pallidotomy for dystonia have reported excellent results with a BFMDRS score improvement of 50%–90%, which is consistent with our findings (74.0% improvement of BFMDRS).^10^, ^12^, ^14^, ^23^, ^28^, ^29^ A recent study by Gross et al reported that bilateral pallidotomy using laser interstitial thermal therapy in two dystonia patients led to a 40.4% and 45.7% BFMDRS score improvement at 1‐year follow‐up.^22^ Contrastingly, Lin et al. reported BFMDRS score improvement of only 13% after bilateral pallidotomy at 1‐year follow‐up in 18 patients with generalized dystonia.^30^ Moreover, significant improvements have been limited to the craniocervical region.^30^ In our study, all BFMDRS subitems of craniocervical region improved with statistical significance. The “trunk” improved with 70.0% improvement without statistical significance. This could be attributed to inadequate statistical power resulting from small sample sizes.

There have been few reports of unilateral pallidotomy for dystonia.^17^, ^26^, ^27^, ^31^, ^32^ Ondo et al. reported that unilateral pallidotomy led to a 50%–73.9% improvement in the BFMDRS score of two and one patients with trauma‐induced and hereditary dystonia respectively.^32^ Alkhani et al. reported an 84% improvement of the Unified Dystonia Rating Scale (UDRS) at the 2‐year postoperative follow‐up in a patient with hemidystonia of unknown origin.^31^ Moreover, Tripathi et al. presented a patient with hemidystonia who underwent unilateral pallidotomy using gamma knife, which achieved a 61% improvement of the UDRS score at the 6‐year follow‐up.^27^ Moreover, we previously reported a patient with blepharospasm who completely improved after unilateral pallidotomy^33^ with the good condition being maintained at the 50‐month follow‐up. We recently reported that unilateral pallidotomy achieved a 47.9% improvement of the TWSTRS score at the 6‐month follow‐up. GPi‐DBS is widely used as a surgical intervention for patients with bilateral, but not unilateral, craniocervical, or truncal dystonia.^3^, ^4^, ^5^ However, few studies have reported the effects of unilateral pallidotomy or GPi‐DBS for patients with midline dystonia distribution using objective evaluation scales.^17^, ^26^, ^33^ Escamilla‐Sevilla et al. reported a patient with cervical dystonia who underwent unilateral GPi‐DBS, which achieved a 94.1% improvement of the BFMDRS score.^34^ Torres et al. reported that unilateral GPi‐DBS in a patient with cervical dystonia achieved a 60% improvement in the TWSTRS score at the 10‐month follow‐up.^35^ These clinical findings are consistent with our findings where all midline BFMDRS subitems significantly improved from 43.2% to 60.3%.

This is the first study to report complications of parkinsonism induced by pallidotomy for dystonia. Recent studies have reported GPi‐DBS‐induced parkinsonism, including bradykinesia, postural instability, and gait disturbance.^36^, ^37^, ^38^, ^39^ However, there has been no report of pallidotomy‐induced parkinsonism in patients with dystonia, which could be due to the limited studies on pallidotomy safety for patients with dystonia. Many studies have reported the use of pallidotomy for patients with PD. There have been several reports of pallidotomy worsening pre‐existing parkinsonism, including bradykinesia, postural instability, and gait disturbance.^40^, ^41^, ^42^ We found that bilateral pallidotomy induced postural instability and gait disturbance with an unacceptable rate of onset (40%). All these cases did not improve spontaneously and were refractory to levodopa. Two of these cases involved severe postural instability and gait disturbance, which resulted in frequent falling and severely worsened quality of life. Consistent with our findings, Parkin et al. reported deterioration of freezing of gait in patients with PD who underwent bilateral pallidotomy after analyzing 51 and 52 unilateral and bilateral pallidotomies, respectively.^41^

Previous studies have indicated that bilateral pallidotomy has a much higher risk than unilateral pallidotomy. In our study, unilateral pallidotomy did not induce dysarthria and dysphagia. One patient who underwent bilateral pallidotomy presented with severe dysarthria and dysphagia without parkinsonism. Except for parkinsonism, the remaining adverse events were very mild, which did not interfere with daily activities. The adverse events associated with unilateral pallidotomy were considered acceptable. However, there was an unacceptably high incidence of postural instability and gait disturbance, which was refractory to levodopa and unpredictable, after bilateral pallidotomy. Regarding avoiding parkinsonism associated with bilateral pallidal interventions, we recently reported that the pallidothalamic tract could be a possible treatment target for dystonia.^43^ Combined unilateral pallidotomy with contralateral pallidothalamic tractotomy achieved 74.3% improvement of the BFMDRS score in 11 patients with dystonia but did not induce parkinsonism.^43^ There is a need for further studies to confirm the safety and efficacy of this combined ablative treatment. Recent reports have indicated that bilateral GPi‐DBS induced parkinsonism including postural instability and gait disturbance in patients with dystonia.^36^, ^39^, ^44^ However, adjustments of stimulation parameters can relieve stimulation‐induced parkinsonism. When performing bilateral pallidal intervention, we consider DBS should be selected to avoid irreversible severe parkinsonism.

Cerebral infarction in the posterior limb of internal capsule (PLIC) adjacent to the pallidotomy lesion developed after a few postoperative weeks in four (4.5%) patients, which caused temporary hemifacial palsy or hemiparesis. There have been few reports of delayed infarction associated with pallidotomy, including 3 (6%) cases reported by Lim et al after radiofrequency pallidotomy.^45^ All infarctions were on the PLIC adjacent to the pallidotomy lesion, which occurred on postoperative days 10, 51, and 117, respectively.^45^ Moreover, they reported that a history of vascular disease could increase the risk for post‐pallidotomy delayed capsular infarction. In our study, two patients with delayed infarction had a previous history of cerebral infarction and coronary artery disease; moreover, all infarctions developed after about one postoperative month. The detailed mechanism of delayed injury to adjacent tissue remains unclear. Lesion involvement of perforating arteries arising from the middle cerebral artery to supply blood to the PLIC may contribute to the post‐pallidotomy onset of cerebral infarction in the PLIC.

This study has several limitations. First, open‐label and retrospective studies have observer and patient bias. Subtle neurological deficits may be missed given the lack of preoperative unified evaluation procedures. Second, given the limited number of patients who underwent bilateral pallidotomy, we could not determine the effect of bilateral pallidotomy on focal affected regions other than the neck. Third, recommended dystonia rating scales for each focal dystonia (e.g. eyes: Blepharospasm Disability Index, neck: Toronto Western Spasmodic Torticollis Rating Scale) was not performed in this study.^46^ Alternatively, subscale of BFMDRS‐MS was used to evaluate each focal dystonia, which is not strictly accurate compared to recommended dystonia rating scales. Fourth, the overall follow‐up period was short. The effects of pallidotomy for dystonia have the possibility of waning benefits in the long run.

In conclusion, we found that unilateral and bilateral pallidotomy provided 51.8% and 74.0% improvement, respectively, in the total BFMDRS score. Bilateral pallidotomy often induced medically refractory parkinsonism, which are newly reported adverse effects of bilateral pallidotomy. The advent of MRI‐guided focused ultrasound procedures, which do not require skin incision, has prompted the reappraisal of ablative treatments. Unilateral radiofrequency pallidotomy remains a viable treatment option for patients with some forms of dystonia. Bilateral pallidotomy cannot be recommended due to unacceptably high complication rates.

## Authors’ Contributions

SH and TT conceived the study. SH, AF, NT and TT collected data. SH and TT designed the study. AF performed the statistical analysis. SH analyzed the data. SH and TT interpreted the data. SH performed literature search and made figures.

## Conflict of Interest

The authors declare no conflicts of interest.

## Data Sharing Statement

The deidentified participant data will be available for a qualified investigator. Please email the corresponding author for more information.
